# Rational Classification of Portal Vein Thrombosis and Its Clinical Significance

**DOI:** 10.1371/journal.pone.0112501

**Published:** 2014-11-13

**Authors:** Jingqin Ma, Zhiping Yan, Jianjun Luo, Qingxin Liu, Jianhua Wang, Shijing Qiu

**Affiliations:** 1 Department of Interventional Radiology, Zhongshan Hospital, Fudan University, Shanghai, China; 2 Bone and Mineral Research Laboratory, Henry Ford Hospital, Detroit, Michigan, United States of America; University of Modena & Reggio Emilia, Italy

## Abstract

Portal vein thrombosis (PVT) is commonly classified into acute (symptom duration <60 days and absence of portal carvernoma and portal hypertension) and chronic types. However, the rationality of this classification has received little attention. In this study, 60 patients (40 men and 20 women) with PVT were examined using contrast-enhanced computed tomography (CT). The percentage of vein occlusion, including portal vein (PV) and superior mesenteric vein (SMV), was measured on CT image. Of 60 patients, 17 (28.3%) met the criterion of acute PVT. Symptoms occurred more frequently in patients with superior mesenteric vein thrombosis (SMVT) compared to those without SMVT (p<0.001). However, there was no significant difference in PV occlusion between patients with and without symptoms. The frequency of cavernous transformation was significantly higher in patients with complete PVT than those with partial PVT (p<0.001). Complications of portal hypertension were significantly associated with cirrhosis (p<0.001) rather than with the severity of PVT and presence of cavernoma. These results suggest that the severity of PVT is only associated with the formation of portal cavernoma but unrelated to the onset of symptoms and the development of portal hypertension. We classified PVT into complete and partial types, and each was subclassified into with and without portal cavernoma. In conclusion, neither symptom duration nor cavernous transformation can clearly distinguish between acute and chronic PVT. The new classification system can determine the pathological alterations of PVT, patency of portal vein and outcome of treatment in a longitudinal study.

## Introduction

Portal vein thrombosis (PVT) is still considered as a rare disease since the primary information has been derived from clinical series and case reports [1], [2]. Recently, Ogren et al. [3] reported that PVT was found in 254 cases of 23,796 autopsies, suggesting that the prevalence of PVT is about 1% of the general population. The lower finding rate of PVT may be related to the difficulty of diagnosis because a large number of patients remain asymptomatic [4]–[6]. With development of imaging techniques (contrast enhanced ultrasound, spiral CT-scan and high definition MRI, etc), PVT may no longer be a rare disease as expected before. PVT has been defined as acute and chronic entities [7]. The duration of symptoms and presence of portal cavernoma or complications of portal hypertension have been used to distinguish between acute and chronic PVT. Although not universally accepted, acute PVT is considered as patients with symptoms <60 days prior to diagnosis and in the absence of portal cavernoma and/or portal hypertension [8], [9]. In contrast, chronic PVT is often accompanied with portal cavernoma and portal hypertension, resulting in esophageal varices, ascites and splenomegaly [4], [7]. There is perceptible difference in the treatment of acute and chronic PVT [7], [10]. Recanalization of obstructed veins is often the primary treatment option for acute PVT [7], [11]–[13]. Unlike acute PVT, management of complications of portal hypertension is recommended prior to the recanalization of thrombosed veins for patients with chronic PVT [7], [14], [15].

Nevertheless, there are two reasons to suggest that the classification of acute and chronic PVT is not perfect in clinical practice. First, the duration of symptoms is sometimes not equal to the duration of thrombosis because the thrombus may have been formed long before the onset of symptoms [4], [5]. In this case, chronic PVT is likely to be diagnosed as an acute one. Second, the formation of portal cavernoma is often not associated with the time of thrombosis. Portal cavernoma would develop rapidly (usually as early as a few days after thrombus formation) from pre-existing veins, particularly in patients with complete thrombosis [4], [7], or not occur long after PVT (e.g. with symptoms >60 days), particularly in patients with partial thrombosis [16], [17]. Thus, it is also illogical to use the presence of portal cavernoma as a criterion to distinguish between acute and chronic PVT.

The purposes of this study were to define the rationality of dividing PTV into acute and chronic types and attempted to find out an appropriate classification for PVT.

## Materials and Methods

### Patient selection

Between January 2005 and November 2012, 71 consecutive patients with PVT were recruited in this study. The following patients were excluded from the study: 1) aged >75 years; 2) without thrombus in portal vein; 3) with the conditions of tumorous thrombosis, severe hepatic encephalopathy or cardiopulmonary co-morbidity and 4) previously received intravenous intervention or surgical portosystemic shunt. The patients with missing data or poor quality CT images were also excluded. After screening, 60 patients (40 men and 20 women) were eligible for this study. The average age was 48.0±13.8 years (range: 16–75 years). Twenty four patients suffered from cirrhosis. The written informed consent to participate in our study and publish these case details was obtained from each adult patient as well as from the guardians on behalf of the children. The study was approved by the Institutional Review Board of Shanghai Zhongshan Hospital.

### Computed tomography

All recruited patients were examined using contrast-enhanced 64-slices spiral computed tomography (CT). CT images were read by two experienced radiologists to identify the location of thrombus and the presence of portal cavernoma. The degree of vein occlusion, including portal vein (PV), superior mesenteric vein (SMV) and splenic vein (SV), was measured on CT image using an image analysis program (Image J, NIH). The areas of vein lumen and inside thrombus were measured on the cross-sectional image at the level of the maximum thrombosis. The percentage of lumen occlusion was calculated by the area of thrombus dividing by the area of vein lumen. Maximum lumen occlusion was used for the determination of the severity of portal vein thrombosis (PVT), superior mesenteric vein thrombosis (SMVT) and splenic vein thrombosis (SVT). Thrombosis was arbitrarily defined as complete when the vein lumen was occluded for more than 90% [18]. Liver cirrhosis was also diagnosed using CT imaging as reported elsewhere [19].

### PVT Classification

Classification of PVT depended on 3 criteria: 1) onset of symptoms such as abdominal pain or distention, diarrhea, nausea, vomiting, anorexia and fever; 2) development of portal cavernoma; 3) presence of complications of portal hypertension including gastroesophageal variceal bleeding, splenomegaly and ascites. PVT was defined as acute if there was a recent episode of symptoms (<60 days) with no evidence of portal cavernoma and complications of portal hypertension. In contrast, chronic PVT was often asymptomatic, in addition to the presence of portal cavernoma and/or complications of portal hypertension.

### Statistical analysis

Continuous variables were expressed as mean and standard deviation (SD). For continuous variables, the mean values were compared using student *t* test or one-way ANOVA. Mann-Whitney test or Kruskal–Wallis test was used if the variable was not normally distributed. Categorical variables were compared using Fisher exact test. Logistic analysis was used to assess: 1) the likelihood of symptom onset associated with the presence of SMVT; 2) the likelihood of cavernous transformation associated with the degree of PVT and 3) the risk of complications of portal hypertension associated with cirrhosis. The level of statistical significance was accepted at p<0.05.

## Results

### Characteristics of patients at diagnosis of PVT

PVT was present in all 60 patients. In these patients, 21 were associated with SMVT, 3 with SVT and 20 suffered thrombosis in both veins.

**Figure 1:**
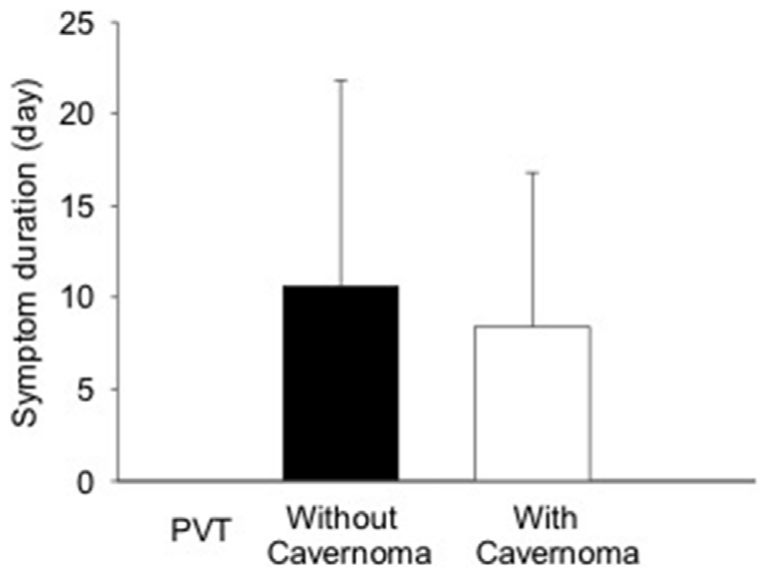
Comparison of symptom duration between PVT patients with and without portal cavernoma. There was no significant difference in symptom duration between two groups.

**Figure 2:**
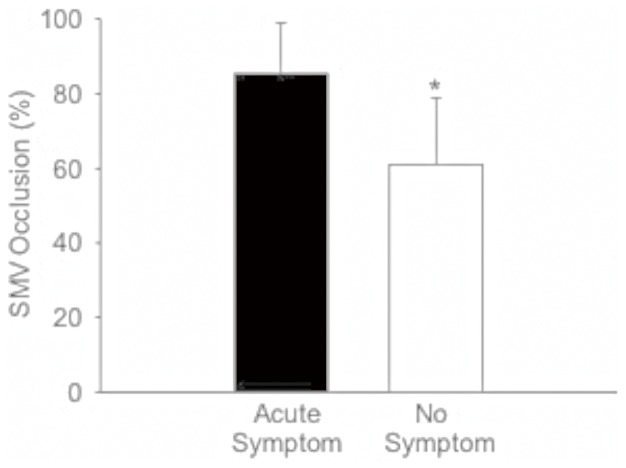
Comparison of superior mesenteric vein (SMV) occlusion between patients with and without recent episode of symptoms. SMV occlusion in patients with symptoms was significantly higher than those without symptom. *p<0.01.

Of 60 patients, 33 had recent onset of symptoms (symptom duration: 0–37 days prior to diagnosis) and 21 were asymptomatic (Table 1). **In** these 2 groups, the severity of PVT and the presence of portal cavernoma were not significantly associated with symptom onset (Table 1). There was no significant difference in PV lumen occlusion between patients with and without symptoms. In patients with recent onset of symptoms, there was no significant difference in symptom duration between subjects with and without portal cavernoma (mean duration: 8.40 *vs* 10.6 days)(Fig 1). However, symptoms occurred significantly more frequent in patients with SMVT than those without SMVT (p<0.001)(Table 1). In patients with SMVT, the SMV lumen occlusion was significantly increased in the subjects with recent symptoms (SMV occlusion 85.5±13.3%) compared to those without symptom (SMV occlusion 65.8±27.9%)(p<0.05)(Fig 2). Logistic analysis demonstrated that the likelihood of symptom onset was 13.3 times (Odds ratio  = 13.3, 95%CI: 3.07–57.9, p<0.001) higher in patients with SMVT than those without SMVT. According to the criteria of classification, 17 patients (28.3%), who experienced recent onset of symptoms but had no portal cavernoma and complication of portal hyertension, were defined as acute PVT and 43 others (71.7%) were defined as chronic PVT (Table 2).

**Table 1:** Relationship of recent symptom onset to the severity of PVT and the presence of MVT and cavernoma.

	Symptom (<60 days)	No Symptom	P
	n = 33	n = 21	
PVT			
Partial	22 (66.7)	15 (71.4)	0.772
Complete	11 (33.3)	6 (28.6)	
SMVT			
No	3 (9.09)	12 (57.1)	<0.001
Yes	30 (90.9)	9 (42.9)	
Cavernoma			
No	19 (57.6)	9 (42.9)	0.403
Yes	14 (42.4)	12 (57.1)	

Data expressed as number (percent).

**Table 2:** Classification of 60 patients with PVT.

	Number	Symptoms	Cavernoma and/or portal
Acute	17	<60 days	No
Chronic	16	<60 days	Yes
	6	≥60 days	Yes
	21	No	Yes

### Relationship between severity of PVT and portal cavernoma

**Figure 3:**
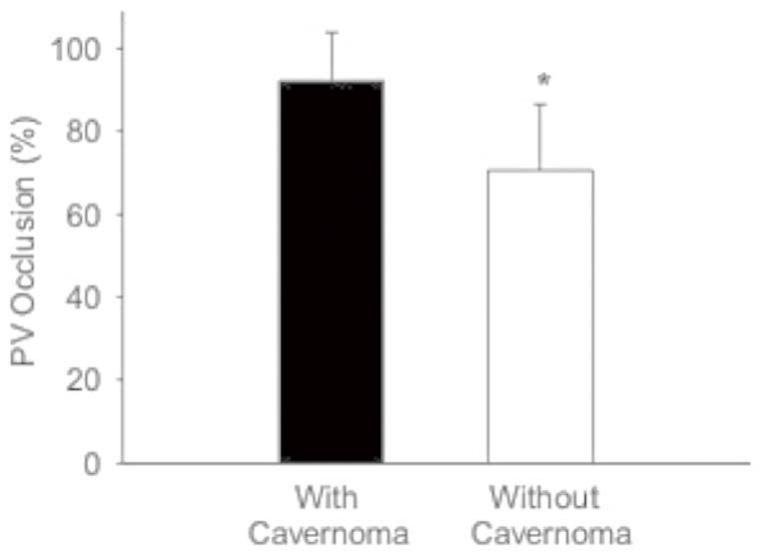
Comparison of portal vein (PV) occlusion between patients with and without cavernoma. PV occlusion in patients with cavernoma was significantly higher than those without cavernoma. *p<0.001.

The mean occlusion of PV was 81.1±17.1% (range: 8–100%) in 60 patients. Twenty two patients had complete PVT, and 20 (90.9%) of them were associated with portal cavernoma. Conversely, in 38 patients with partial PVT, only 11 (28.9%) were associated with portal cavernoma. The frequency of cavernous transformation was significantly higher in patients with complete PVT than those with partial PVT (p<0.001, Table 3). The PV lumen occlusion was significantly higher in patients with portal cavernoma than those without cavernoma (p<0.001, Fig 3). Logistic analysis demonstrated that the likelihood of cavernous transformation was 24.5 times (Odds ratio  = 24.5, 95%CI: 4.89–123, p<0.001) higher in patients with complete PVT than those with partial PVT.

**Table 3:** Relationship between severity of PVT and cavernous transformation.

	Partial PVT	Complete PVT	P
	n = 38	n = 21	
Cavernoma			
No	27 (71.7)	2 (9.09)	<0.001
Yes	11 (28.9)	20 (90.9)	

Data expressed as number (percent).

**Figure 4:**
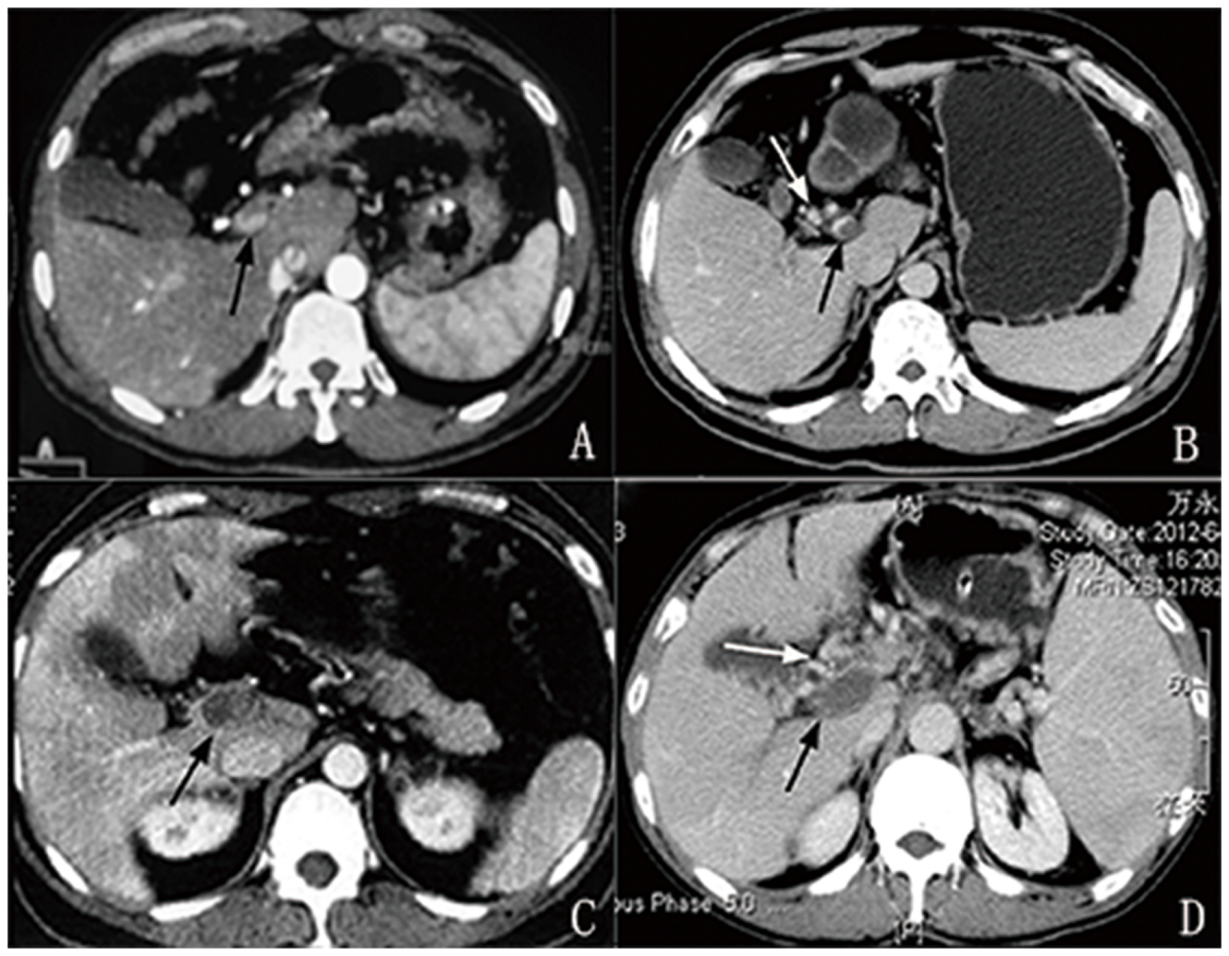
Cross-sectional CT image of portal vein thrombosis and cavernoma: A) partial PVT (black arrow) without cavernoma; B) partial PVT (black arrow) with cavernoma (white arrow); C) complete PVT (black arrow) without carvernoma and D) complete PVT (black arrow) with cavernoma (white arrow).

In this combination between severity of thrombosis and presence of portal cavernoma, four types of PVT were seen (Fig 4): **1) partial PVT without cavernoma (27/60, 45.0%); 2) partial PVT with cavernoma (11/60, 18.3%); 3) complete PVT without cavernoma (2/60, 3.33%) and 4) complete PVT with cavernoma (20/60, 33.3%).**

### Relationship between cirrhosis and complications of portal hypertension

Twenty seven patients had complications of portal hypertension, including 26 gastroesophageal variceal bleeding and 1 ascites. Complications were significantly associated with cirrhosis (p<0.001) rather than the severity of PVT and presence of cavernoma (Table 4). In 27 patients with complications, 19 (70.4%) suffered from cirrhosis. However, in 33 patients without complications only 6 (18.2%) had cirrhosis. The patients with cirrhosis had a 10.7 times (Odds ratio  = 10.7, 95%CI: 3.19–35.9, p<0.001) higher risk of complications of portal hypertension compared to those without cirrhosis.

**Table 4:** Relationship of complications of portal hypertension with PVT, cavernoma and cirrhosis.

	Complications	No complications	P
	n = 27	n = 33	
PVT			
Partial	18 (66.7)	20 (60.6)	0.789
Complete	9 (33.3)	13 (39.4)	
Cavernoma			
No	11 (40.7)	18 (54.5)	0.312
Yes	16 (59.3)	15 (45.5)	
Cirrhosis			
No	8 (29.6)	27 (81.8)	<0.001
Yes	19 (70.4)	6 (18.2)	

Data expressed as number (percent).

**Figure 5:**
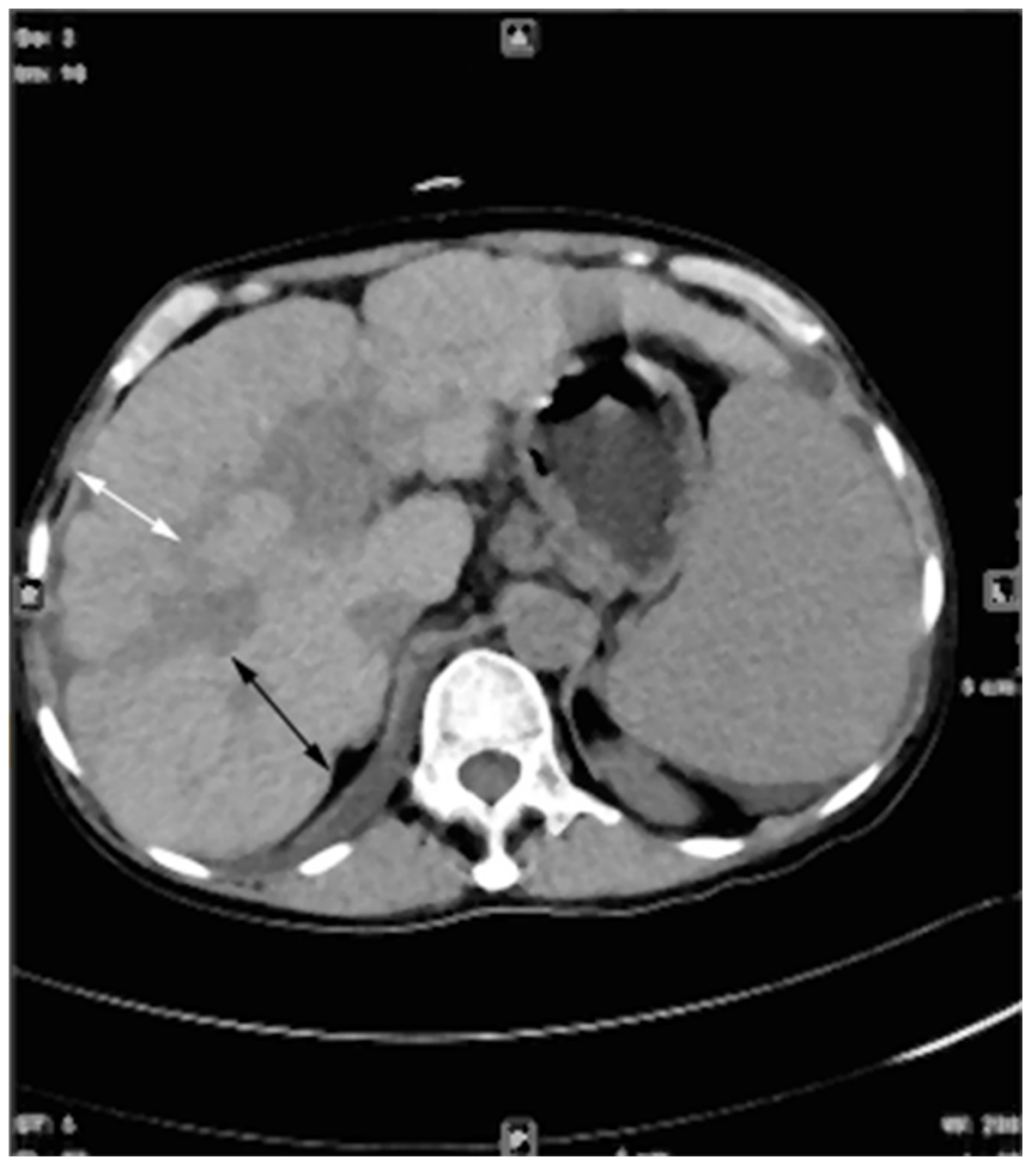
Cirrhotic morphology in CT imaging. Portal venous phase CT scan shows nodularity on the liver surface and heterogeneity of the liver parenchyma. A ratio of transverse caudate lobe width (black arrow) to right lobe width (white arrow) is greater than 0.65. Splenomegaly was present.

In this study, cirrhosis was diagnosed from liver morphology in CT imaging (Fig 5). The cirrhosis was characterized by surface nodularity and heterogeneity of liver parenchyma. A ratio of transverse caudate lobe width to right lobe width greater than or equal to 0.65 was a positive indicator for the diagnosis of cirrhosis [19]. Splenomegaly was often found in patients with cirrhosis.

## Discussion

Acute and chronic PVT, as the name implies, are distinguished by the time of thrombus presence. However, it is difficult to determine the exact time of thrombus formation. Instead, the duration of symptoms and the aftermaths of portal vein occlusion, such as portal cavernoma and portal hypertension, have been used as the criteria to distinguish between acute and chronic PVT [4], [7], [8], [20]. The patients who developed symptoms <60 days and had no evidence of portal cavernoma and portal hypertension can be diagnosed as acute PVT, while the others are considered as chronic PVT [9], [21], [22]. Based on our results and literature review, we assume that the symptoms are unreliable for the classification of PVT due to the following reasons. First, the symptom onset does not occur at the same time as the initiation of PVT. In other words, the duration of symptoms is not equal to the duration of thrombosis; the former is frequently shorter than the latter. Additionally, many patients with PVT may remain asymptomatic for a long period of time [21]. In our study, 35% (21/60) of patients with PVT were asymptomatic. There is no consensus about the classification of asymptomatic PVT [10], [23], [24]. Most authors consider asymptomatic PVT as chronic PVT [10], [21]. However, there is no evidence to confirm that PVT in asymptomatic patients exists longer than that in patients with symptoms. Last but not least, our results showed that symptom onset was not associated with the severity of PVT but significantly related to the severity of SMVT. The typical presentation of acute PVT is with acute abdomen [22]. It is well known that the symptoms of acute abdomen are associated with bowel ischemia, which is caused by the occlusion of superior mesenteric vein [13], [25]. Since the occlusion of portal vein would not directly cause severe bowel ischemia, the symptom onset seems unlikely to be **the** result of PVT.

When PVT in its acute stage remains unsolved, collateral vessels will appear around portal vein from a few days to weeks and eventually become a spongelike portal cavernoma [26]. The number, size, and location of collaterals are variable among different patients [21], [22]. At present, many authors ascertain chronic PVT by the presence of portal cavernoma [6], [7], [10]. However, the portal cavernoma in some patients does not occur a long time after acute event [16], [26], [27]. De Gaetano et al. [26] followed up 131 PVT patients up to 6 weeks and found that 56 (42.7%) patients had no portal cavernoma during the whole follow-up period, 66 (50.4%) showed a cavernoma and 9 (6.9%) had no cavernoma at the first examination. In these 9 patients, cavernoma occurred within 6–20 days after thrombus formation. Luca et al. [27] reported that 14% of patients with partial PVT developed to complete PVT in two years, but none of them showed portal cavernoma. Our study demonstrated that the development of portal cavernoma was not correlated with the duration of symptoms. In patients with recent onset of symptoms, 42% were associated with portal cavernoma. The symptom duration for these patients was 2 days shorter than the patients without portal cavernoma, although the statistical significance was not reached. Portal cavernoma may be found very shortly after symptom onset (0–2 days) in some patients. These data suggest that cavernous transformation of portal vein may occur at the early stage of PVT. Accordingly, portal cavernoma is inappropriate to be used for the diagnosis of chronic PVT.

In contrast to symptom onset, cavernous transformation was significantly associated with the severity of PVT. We found that >90% of patients with complete PVT were accompanied with portal cavernoma, which was remarkably more than 35% in patients with partial PVT. Even in patients with partial PVT, the PV lumen occlusion was significantly higher in subjects with portal cavernoma (PV occlusion  = 79.5%) than those without cavernoma (PV occlusion  = 68.7%). The likelihood of cavernous transformation in patients with complete PVT (PV occlusion >90%) was 24 times higher than patients with partial PVT (PV occlusion ≤90%).

There are several diverse classifications based on the degree and extension of PVT [28]–[32] due to their close association with the outcome of treatment [33]. Complete PVT would cause cessation of portal blood flow, resulting in liver to lose about two thirds of its blood supply. However, this condition is usually well tolerated and patients are often asymptomatic [21]. It is probably due to the rapid development of portal cavernoma that is composed of numerous hepatopetal collateral vessels located in the hepatic hilum [34]. These collateral vessels connect the two patent portions proximately and distally to the thrombus and partially supplement the loss of portal vein's contribution to liver blood flow. This compensatory mechanism may make the obstructed portal vein lose its own function and eventually become a thin, fibrotic cord [6], [7]. At this stage, portal hypertension may occur [35], [36]. The large portal cavernoma may also compress the pliable common bile duct, resulting in the formation of portal biliopathy [37]. Therefore, the changes in thrombus and portal cavernoma should be both examined in the treatment of PVT.

The significant relationship between the severity of PVT and portal cavernoma has made us create a new classification system, in which PVT is classified into 4 types according to the degree of portal vein occlusion and the association with portal cavernoma. Type I is partial PVT without cavernoma, type II is partial PVT associated with cavernoma, type III is complete PVT without cavernoma and type IV is complete PVT associated with cavernoma. In our cohort, 51.6% of PVT patients (complete 33.3% and partial 18.3%) were associated with cavernoma and others (complete 3.33% and partial 45.0%) were not associated with cavernoma, suggesting that cavernous transformation is significantly correlated with the severity of portal vein occlusion. There are **several** advantages of this classification. First, it is easy to do the classification because both PVT and portal cavernoma can be clearly identified by CT or MRI imaging [10], [38], [39]. Every patient can be clearly classified into a certain type with no ambiguous variable. In addition, the degree of portal vein occlusion can be quantified on CT imaging, which can detect little changes in the severity of PVT. Second, this classification is able to demonstrate the pathological alterations of PVT, patency of portal vein and outcome of treatment. For example, this classification can ascertain whether the partial PVT become worsened, improved or stable with time [27], and can also detect the improvement and recurrence of PVT after anticoagulant and interventional therapies [18], [40]–[43]. Third, optimal treatment can be determined based on this classification, at least during a certain period. For example, cavernoma was once seen as a contraindication of transjugular intrahepatic portosystemic shunt (TIPS) in patients with PVT [44]. With the development of stent design and surgical technique, cavernoma is no longer a contraindication of TIPS, but it does increase the technical difficulty of the procedure [9].

In this study, 27 patients (45%) suffered from complications of portal hypertension, 26 with gastroesophageal variceal bleeding and 1 with severe ascites. The prevalence of complications was 30% in patients without cirrhosis, but increased to 70% in patients with cirrhosis. The risk of complications in patients with cirrhosis was approximately 11 times higher than patients without cirrhosis. Nevertheless, the complications of portal hypertension were not significantly associated with the degree of thrombosis and the presence of portal cavernoma. Likewise, Amarapurkar et al. [45] reported recently that portal vein occlusion is an uncommon cause of portal hypertension in adults in India. Luca et al. [27] also reported that the progression of partial PVT did not increase the risk of complications resulting from portal hypertension. Liver cirrhosis has been confirmed as a critical factor contributing to the complications of portal hypertension [46]. Ponziani [47] proposed that PVT development is not only a matter of impaired blood flow or pro-coagulation tendency, but also a consequence of the worsening in portal vein outflow due to increased hepatic resistance in cirrhotic liver. Since many cirrhotic patients may have had portal vein hypertension before the occurrence of PVT, the complications in these patients **are** likely to be independent of the severity of PVT.

Our study had some limitations. The major limitation is that this was a cross-sectional retrospective study, which could not ascertain the value of the new classification in the evaluation of PVT progress and treatment outcome. Additionally, the study might not have adequate power to detect significant correlation between the complications of portal hypertension and the severity of PVT and portal cavernoma.

In conclusion, recent onset of symptoms was usually initiated by severe SMVT rather than PVT and portal cavernoma. Neither symptom duration nor cavernous transformation is appropriate to be used as a criterion to distinguish between acute and chronic PVT. It seems rational to classify PVT based on PVT severity and cavernous transformation of portal vein, which is suitable to be used in the longitudinal study to determine the pathological alterations of PVT, patency of portal vein and outcome of treatment.
